# Editorial: Understanding brain mechanisms underpinning physical movement and exercise

**DOI:** 10.3389/fnrgo.2022.1014597

**Published:** 2022-09-16

**Authors:** Wei-Peng Teo, Stephane Perrey

**Affiliations:** ^1^Physical Education and Sports Science Academic Group, National Institute of Education, Nanyang Technological University, Singapore, Singapore; ^2^EuroMov Digital Heath in Motion, Univ Montpellier, IMT Mines Ales, Montpellier, France

**Keywords:** neuroimaging, NIRS, transcranial magnetic stimulation, transcranial direct current stimulation, neuroplasticity

## Introduction

It is well-accepted that physical activity and exercise, exert a strong positive influence over the central nervous system. As such, there is significant interest on understanding how specifically exercise influences neuroplasticity, and how the brain controls movement to perform daily activities. To truly understanding these mechanisms, neuroimaging techniques such as magnetic resonance imaging have revealed some insights on how the brain controls motor function and responses to exercise. However, these techniques may be limited in terms of their temporal resolution and ecological validity in measuring brain responses to movement and exercise. Now, recent advances of neuroimaging devices such as portable electroencephalography, functional near-infrared spectroscopy (fNIRS), and non-invasive brain stimulation techniques such as transcranial direct current stimulation (tDCS) and transcranial magnetic stimulation (TMS) can be used to study a broader range of dynamic movements and central changes associated with physical exercise. Portable neuroimaging methods can be applied concurrently with a motor task or exercise to understand its associated neural response, while the application of non-invasive brain stimulation can help to establish causality by experimentally-induced facilitation or inhibition of specific neural networks. Therefore, we hosted a special Research Topic issue for Frontiers in Neuroergonomics that focused on brain mechanisms underpinning physical movement and exercise. In total, 8 papers were accepted totalling 31 authors that covered three main domains: (1) Methods to elucidate fine motor control, (2) Exercise-related brain adaptations, and (3) Prospective considerations.

## Methods to elucidate neural control of ecological movement

While the role of the primary motor cortex in fine motor movement and handedness are well-established (Garcia et al.), less is known about the associated motor areas, such as the premotor areas, on fine motor skills. Indeed most of our understanding of the premotor area comes from observing patients with brain lesions with associated fine motor skills deficits. To truly determine the role of the premotor area on fine motor control, Fleischmann et al. used navigated TMS to induce virtual lesions at discrete timings during an established tracing task to the premotor area in 10 healthy subjects. What they found were significantly greater errors made in all subjects when TMS pulses were applied 120 and 140 ms to the premotor area prior to a turning point on the tracing task as compared to motor cortex or dorsolateral prefrontal cortex TMS.

FNIRS acts similarly to functional MRI to determine changes in oxyhemoglobin concentration during a specific task. As new fNIRS devices become smaller and more portable, its greatest strength lies in its ability to measure reliable changes in oxyhemoglobin during an ecologically relevant task (e.g., walking, cycling, or balancing activities) compared to MRI. As an example, Seidel-Marzi et al. used a portable 22-channel fNIRS system during a slackline stationary balance and walking task in advanced slackliners to determine differences in activation of several brain motor regions in the left and right hemispheres. They showed an increase in hemodynamic response of the sensorimotor brain regions during both stationary standing and walking on a slackline, with no difference in increased hemodynamic response between both conditions. The authors attributed this lack of difference between standing and walking on a slackline to be due to similarities in task complexity and demand.

In this sense, both publications highlighted the use of advanced neuroimaging techniques to broaden our understanding of human motor control of the upper and lower limbs. The novelty of these studies stem from the fact that TMS and fNIRS techniques were employed in motor tasks that were not physically constraint (i.e., lying down in an MRI scanner) and used a motor task that was ecologically valid to the research question. Moving forwards, future studies should aim to employ more contextualized motor tasks to truly understand the implicit nature of fine motor control.

## Understanding exercise-related adaptations in the brain

Exercise is known to elicit physiological benefits to the metabolic, cardiovascular, musculoskeletal, and even the central nervous systems. Probably one of the most well-studied mechanism underpinning exercise-induced neuroplasticity, is the role of brain-derived neurotrophic factor (BDNF) on brain structure and function. To date, there is overwhelming evidence that both aerobic (Wang et al., 2022) and resistance exercise (Chow et al., 2021) elicit a cascade of factors that promote the release of peripheral BDNF. While this may be true, Nicolini and Nelson provided a comprehensive review on the methodological and non-methodological factors that may affect the reproducibility of measuring peripheral BDNF concentration. Most notably, all these factors are known to affect plasma and serum BDNF expression differently, which may lead to complications in comparing results from different studies that use seemingly similar protocols and equipment.

Apart from understanding how exercise influences neuroplasticity at a molecular level, exercise-induced TMS-evoked changes have been well-established (Turco and Nelson). In their review, Turco and Nelson provided a thorough overview of the TMS-related changes associated with single- and paired-pulse paradigms following acute and chronic aerobic exercise. Similar to studies assessing BDNF changes associated with exercise, it is apparent that TMS measures are subject to methodological and non-methodological factors that may influence the overall measurement outcomes. Lifestyle factors such as age, baseline aerobic fitness, menstrual cycle and regular physical activity are known markers that may directly influence TMS outcomes. Together with methodological considerations such as timing of TMS measure, coil measures and diurnal changes, future implementation of TMS will need to consider both individual and protocol factors to produce reliable and reproducible TMS outcomes within and between studies.

To understand the relationship between brain adaptations, aerobic fitness, and mental health outcomes, Crum et al. provided evidence for an inverse association between the inferior frontal gyrus activation using fNIRS and greater symptoms of depression in 106 adult subjects. Furthermore, they showed that higher aerobic fitness levels were positively associated with greater changes in the right rostral prefrontal cortex. While the findings from Crum et al. were largely associative, their findings suggest a mechanistic link between physical fitness and brain function, which underpins cognitive behavior and mood.

## Prospective considerations

Thus far, some evidence provided supports the role of portable neuroimaging (i.e., fNIRS) and non-invasive brain stimulation (i.e., TMS) to understand neuroplasticity. These studies often employ a randomized controlled study design with adequate sample size to detect differences between the intervention and control. Pate and McCambridge however, propose an interesting concept of a single-subject design when examining the effects of non-invasive brain stimulation. Such a study design, also known as single-case studies, are often used in clinical research whereby a thorough consideration of the protocol and measures are used to implement both the intervention and control in a single participant, or in a group of participants but assessed individually. The authors however stress that key challenges of using such a research design are the lack of generalizability particularly in conditions that are heterogeneous (i.e., stroke and dystonia) and subjective interpretation of the results.

In addition, Jaberzadeh and Zoghi proposed the role of interventional non-invasive brain stimulation methods such as tDCS to understand the role of exercise on brain adaptations. While there is already rich literature on the use of tDCS in improving physical performance, the role of tDCS in understanding exercise fatigue and post-exercise brain adaptations are still relatively unknown. Jaberzadeh and Zoghi proposed a framework in which exercise-induced central fatigue may stem from, and tDCS may represent a viable and safe option to counteract post-exercise central fatigue.

In summary, there are still many unknowns when it comes to understanding exercise-induced brain adaptations and more needs to be done to thoroughly elucidate the mechanisms that underpin exercise adaptations on the central nervous system. The key challenges now are to determine dose-response relationships between exercise parameters and psychophysiological outcomes across various populations. Finally, with newer and lighter neuroimaging and neurostimulation systems being developed, it is essential to move from traditional neuroimaging paradigms to newer ecologically-valid motor task to understand neural control of movement.

## Author contributions

SP drafted the Editorial. W-PT reviewed and provided the input. Both authors contributed to the article and approved the submitted version.

## Conflict of interest

The authors declare that the research was conducted in the absence of any commercial or financial relationships that could be construed as a potential conflict of interest.

## Publisher's note

All claims expressed in this article are solely those of the authors and do not necessarily represent those of their affiliated organizations, or those of the publisher, the editors and the reviewers. Any product that may be evaluated in this article, or claim that may be made by its manufacturer, is not guaranteed or endorsed by the publisher.
